# Risk of diabetic ketoacidosis caused by sodium glucose cotransporter-2 inhibitors in patients with type 1 diabetes: a systematic review and network meta-analysis of randomized controlled trials

**DOI:** 10.3389/fendo.2024.1453067

**Published:** 2025-01-31

**Authors:** Ying Liu, Shiwen Yang, Aidou Jiang, Dan Zou, Zhaoyang Chen, Na Su

**Affiliations:** ^1^ Department of Pharmacy, West China Hospital, Sichuan University, Chengdu, China; ^2^ Department of Pharmacy, Jiangxi Mental Health Center, Nanchang, China; ^3^ West China School of Pharmacy, Sichuan University, Chengdu, China

**Keywords:** sodium-glucose-cotransporter-2 (SGLT2) inhibitors, the risk of diabetic ketoacidosis, type 1 diabetes mellitus, network meta-analysis, placebo control

## Abstract

**Background:**

The benefits of sodium-glucose-cotransporter-2 (SGLT2) inhibitors in the treatment of type 1 diabetes mellitus (T1DM) have been demonstrated, but the occurrence of diabetic ketoacidosis (DKA) limits their use. The risk of DKA associated with different doses of SGLT2 inhibitous in the treatment of T1DM is unknown. We conducted a network meta-analysis to evaluate the incidence of DKA at different doses in the treatment of T1DM.

**Methods:**

We searched electronic databases and clinical trial registries, including PubMed, Embase (Ovid SP), the Cochrane Central Register of Controlled Trials (Ovid SP), and ClinicalTrials.gov, for randomized controlled trials (RCTs) concerning SGLT2 inhibitors in patients with T1DM from inception to December 2023. Literature screening, quality assessment and data extraction were carried out independently by 2 researchers based on the inclusion and exclusion criteria, and statistical analysis was performed using Stata 15.1 software and R 4.1.3.

**Results:**

Nineteen clinical studies and one clinical trial were ultimately included. The study involved five different SGLT2 inhibitors. The incidence of DKA in dapagliflozin 5 mg (OR: 2.57, 95% CI: 1.04 to 6.33; P<0.00001), empagliflozin 10 mg (OR: 2.68, 95% CI: 1.11 to 6.49; P<0.00001), sogliflozin 200mg (OR: 4.04, 95% CI: 1.15 to14.18; P<0.00001) and sogliflozin 400mg (OR: 5.96, 95% CI: 2.06 to17.20; P<0.00001) were higher than for the placebo. According to the P scores, SGLT2 inhibitors triggered a lower incidence of DKA than did the placebo. Treatment with 300 mg canagliflozin had the lowest incidence of DKA (P score = 0.8563).

**Conclusion:**

According to our study, 5 mg dapagliflozin,10 mg empagliflozin 200mg sogliflozin and 400mg sogliflozin resulted in DKA when adjunctive insulin was used to treat T1DM. Other SGLT2 inhibitors seem to be safe. However, SGLT2 inhibitors for treating T1DM are off label in China, and adverse reactions should be closely monitored during administration.

**Systematic review registration:**

https://www.crd.york.ac.uk/prospero/#loginpage, identifier CRD42023416227.

## 1 Introduction

Type 1 diabetes mellitus (T1DM) is an endocrine disease caused by the combined action of susceptibility genes and environmental factors, leading to destruction of β cells and a lifelong dependence on insulin therapy. Insulin therapy is the mainstay treatment for people with T1DM (1). However, long-term insulin therapy is accompanied by problems such as hypoglycemia, weight gain, insulin resistance, and increased cardiovascular risk (2). Some noninsulin medications may assist in treating hypoglycemia while potentially counteracting these effects and may have cardiovascular and renal benefits to some extent. Sodium-glucose cotransporter 2 (SGLT2) inhibitors is one of the adjuvant drugs used to treat T1DM.

SGLT2 inhibitors, such as cagliflozin, dagliflozin, and empagliflozin, are a newer class of antihyperglycemic agents that can improve glycemic control in an insulin-independent manner. Adjuvant therapy in patients with T1DM has been shown to reduce HbA1c, improve glucose time in target range, reduce body weight, and improve blood pressure control in several phase III clinical studies (3).In the 2021 Consensus Report on the Management of Type 1 Diabetes in Adults, issued by the American Diabetes Association (ADA) together with the European Association for the Study of Diabetes (EASD), it was stated that SGLT2 inhibitors should be used with caution, with care taken to adjust insulin doses appropriately when using them, starting with a Start with a low dose of SGLT 2 inhibitor and measure blood ketones regularly (4). The Chinese 2021 guideline for the diagnosis and management of type I diabetes suggests that SGLT2 inhibitors may be considered in T1DM with informed consent and a BMI ≥25 kg/m2 with poor insulin control (5). However, the adjunctive use of SGLT 2 inhibitors in adult patients with type 1 diabetes mellitus increases the absolute risk of DKA by approximately 4% per year (6). Because of the increased incidence of DKA the FDA denied marketing authorization for its treatment of T1DM. In 2021, dapagliflozin 5 mg for T1DM indication was removed across the European Medicines Agency and the MHRA (7).

Considering the benefits that SGLT2 inhibitors bring in the treatment of T1DM, the use of these drugs in the treatment of T1DM is currently being explored, despite the possibility that they may lead to an elevated risk associated with DKA.

We conducted a systematic review and network meta-analysis for the risk of DKA associated with SGLT2 inhibitors in the adjuvant treatment of T1DM, with the aim of providing some reference for the safety convenience of clinical use of SGLT2 inhibitors in the adjuvant treatment of T1DM.

## 2 Methods

This network meta-analysis was registered in the PROSPERO database (ID: CRD 42023416227) and conducted according to the Preferred Reporting Items for Systematic Reviews and Meta-Analyses (PRISMA) 2020 guidelines and the statement standard for network meta-analysis (PRISMA-NMA) (**Supplementary Tables 1**, **2**).

### 2.1 Literature search

A systematic review of the literature using PubMed, Embase (Ovid SP), and Cochrane Central Register of Controlled Trials (Ovid SP) for studies was conducted on December 18th, 2023. We also searched ClinicalTrial.gov for ongoing, unpublished studies. Finally, the reference lists of relevant published research investigating the risk of DKA of SGLT2 inhibitors in patients with T1DM were also screened for potentially relevant studies. The key terms searched in this study are based on the PICOS framework, (**Supplementary Table 3**). Duplicate records were removed with EndNote X9.

### 2.2 Study selection

Randomized controlled trials (RCTs) assessing SGLT2 inhibitors in patients with T1DM were evaluated based on the following criteria: (1) Adults (>18 years) with a diagnosis of T1DM. (2) Different medicines must be used in different groups of patients; one group of patients must use SGLT2 inhibitors, and the other group can use other hypoglycemic drugs that are not SGLT2 inhibitors or placebos. (3) Trials reporting on the outcome of the risk of DKA. The exclusion criteria were as follows: 1) pregnant participants; 2) published as abstracts only; and 3) included patients with prediabetes. We had no restriction on language, but all included studies were written in English.

### 2.3 Data extraction and quality assessment

From eligible studies, the first author’s name, publication year, sample size, follow-up length, intervention and comparison, outcomes, and the characteristics of participants were extracted according to a prespecified protocol. Two investigators (YL and AJ) independently performed the literature search, study selection, and extraction of baseline characteristics and outcome measures and cross-checking. Any discrepancy or ambiguity in this process was resolved by discussion with a 3rd reviewer (NS) when necessary. Two independent investigators adjudicated the quality of evidence using the Risk of Bias (ROB 2) tool for randomized trials through six pre-specified domains (8) The evidence quality of collective outcomes was estimated using the Grading of Recommendations Assessment, Development, and Evaluation (GRADE) framework (9). A comparison-adjusted funnel plot with the Egger test was conducted to assess for publication bias (10).

### 2.4 Statistical analysis

We used a fixed-effects model-based frequentist framework with a graph-theoretical method by the netmeta package command R (version 4.1.3) to summarize the risk of DKA between different SGLT2 inhibitors (11, 12). Then, the incidence of DKA in the treatment of T1DM with SGLT2 inhibitors was statistically analyzed by P=score to ranking probability of intervention drugs in main analysis by netmeta package in R. Direct and indirect comparisons were accomplished through the self-programmed routines of Stata and the netmeta package of R (10, 13). Because the outcomes included in this study were dichotomous variables, odds ratios (ORs) were used for effect estimation, and 95% confidence intervals (CIs) and P < 0.05 were used as the criteria for significant differences. We estimated the variance in heterogeneity between studies using the DerSimonian–Laird fixed-effects model. We assessed transitivity using descriptive statistics from studies and population baselines (14).

Publication bias was evaluated utilizing funnel plots and Egger’s test, employing the netmeta package in R software. To ensure the robustness of the final results, four sensitivity analyses were conducted, encompassing the following considerations: 1) exclusion of studies with fewer than 100 participants; 2) exclusion of studies with treatment duration <12 weeks; 3) exclusion of studies without an insulin control;4)this analysis was estimated in a Bayesian framework.

## 3 Results

### 3.1 Characteristics of eligible studies

**Figure 1:**
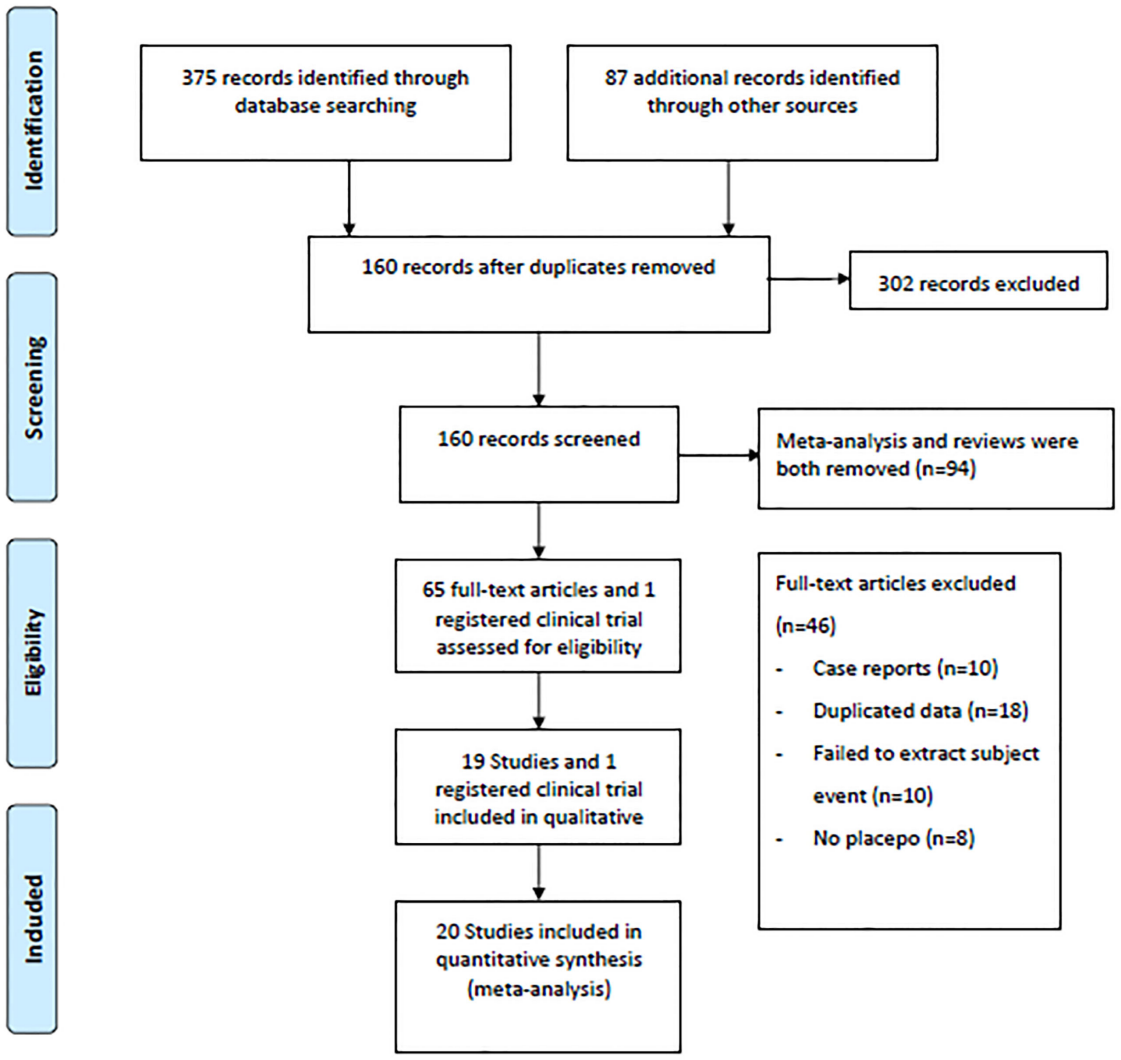
Flow diagram for study identification and inclusion.

A flow diagram illustrating the literature search process is presented in **Figure 1**. After screening 375 articles and 87 additional records identified through other sources, a total of 20 studies conducted between 2015 and 2023 were included in the meta-analysis based on predefined criteria (19 articles and 1 registered clinical trial). These studies involved 7183 patients and 135 patients with DKA (15–33). Of the studies included, 6 were multinational country studies, and all of them were registered and published in English. The baseline characteristics of the included studies are presented in **Table 1**. Among these studies, SGLT2 inhibitors were all adjunctive to insulin for the treatment of T1DM. Nineteen were two-arm studies, and 1 was a single-arm study. Among the twenty studies (the SGLT2 inhibitors retrieved included 1 study about canagliflozin, 6 studies about dapagliflozin, 5 studies about empagliflozin, 2 studies about ipragliflzin and 6 studies about sotagliflozin),14 compared the use of different doses of SGLT2 inhibitors, of which three studies compared sotagliflozin 400 mg/day with 200 mg/day, five studies compared dapagliflozin 5 mg/day with 10 mg/day, and four studies compared empagliflozin 10 mg/day and 25 mg/day. Additionally, two other studies were conducted to compare canagliflozin 100 mg/day with 300 mg/day and ipragliflozin 50 mg/day with 100 mg/day; 19 studies compared SGLT2 inhibitors to placebo. The study included 3497 male participants (48.69%) and 3686 female participants (51.31%), with a mean age of 42.49 years (ranging from 21.70 to 55.00 years); the mean HbA1c was 8.17% (ranging from 6.70 to 9.90%); the baseline mean BMI was 26.98 kg/m^2^ (ranging from 22.20 to 31.80 kg/m^2^); the mean T1DM duration was 19.61 years (ranging from 11.90 to 25.00 years); and the mean treatment duration was 23.1 weeks (ranging from 1.0 to 52.0 weeks). In addition, all trials were funded by pharmaceutical companies.

**Table 1:** Characteristics baseline of randomized controlled trials.

First author	Register number/Trial name	Location	No. of patients(n)	N	Intervention	Age (years) (mean ± SD)	HbA1c (%) (mean ± SD)	BMI (Kg/m^2^) (mean ± SD)	Duration of diabetes (years) (mean ± SD)	Length of follow-up (weeks)
Baker 2019 (15)	NCT02459899	USA	106	35	sotagliflozin 200 mg/day + insulin	47.00 ± 14.00	8.07 ± 0.93	28.00 ± 4.70	23.40 ± 13.20	12 weeks
				35	sotagliflozin 400 mg/day + insulin	44.80 ± 15.40	8.05 ± 0.74	29.40 ± 5.80	24.00 ± 15.00	
				36	placebo + insulin	48.10 ± 11.30	7.95 ± 0.85	31.80 ± 5.80	26.90 ± 13.50	
Bode 2021 (16)	NCT02383940	USA	85	43	sotagliflozin 400 mg/day + insulin	22.80 ± 4.00	9.90 ± 1.40	29.40 ± 7.20	11.90 ± 6.20	12 weeks
				42	placebo + insulin	21.70 ± 3.60	9.70 ± 0.90	26.70 ± 5.00	11.90 ± 5.40	
Buse 2018 (17)	NCT02384941	USA、Canada	793	263	sotagliflozin 200 mg/day + insulin	46.60 ± 13.48	7.61 ± 0.74	29.81 ± 5.686	25.00 ± 13.15	52 weeks
				262	sotagliflozin 400 mg/day + insulin	46.40 ± 13.12	7.56 ± 0.72	29.63 ± 5.297	24.00 ± 12.88	
				268	placebo + insulin	45.20 ± 12.72	7.54 ± 0.71	29.55 ± 5.188	24.20 ± 12.38	
Dandona 2018 (18)	NCT02268214	multinational	778	259	dapagliflozin 5 mg/day + insulin	41.90 ± 14.10	8.53 ± 0.71	28.40 ± 5.80	19.70 ± 12.00	52 weeks
				259	dapagliflozin 10 mg/day + insulin	42.70 ± 14.10	8.52 ± 0.64	28.20 ± 5.20	19.90 ± 11.10	
				260	placebo + insulin	42.70 ± 13.60	8.53 ± 0.67	28.60 ± 5.30	21.20 ± 12.20	
Danne 2018 (19)	NCT02421510	multinational	782	261	sotagliflozin 200 mg/day + insulin	42.30 ± 13.59	7.74 ± 0.81	27.97 ± 5.275	18.20 ± 10.82	52 weeks
				263	sotagliflozin 400 mg/day + insulin	41.70 ± 13.23	7.71 ± 0.82	27.85 ± 4.921	18.90 ± 11.18	
				258	placebo + insulin	39.70 ± 13.42	7.79 ± 0.88	27.50 ± 5.170	18.10 ± 10.72	
Garg 2017 (20)	NCT02531035	multinational	1402	699	sotagliflozin 400 mg/day + insulin	43.30 ± 14.20	8.26 ± 0.96	28.29 ± 5.13	20.50 ± 12.40	24 weeks
				703	placebo + insulin	42.40 ± 14.00	8.21 ± 0.92	28.10 ± 5.18	19.60 ± 12.10	
Garcia-Tirado 2022 (21)	NCT04201496	USA	35	18	empagliflozin 5mg/day + insulin	40.00 ± 14.00	6.70 ± 1.00	30.00 ± 6.00	21.00 ± 13.00	8 weeks
				17	placebo + insulin	42.00 ± 13.00	7.10 ± 1.00	29.00 ± 5.00	21.00 ± 13.00	
Henry 2015-1 (22)	NCT01498185	USA	42	14	dapagliflozin 5 mg/day + insulin	34.80 ± 14.00	8.50 ± 0.78	23.40 ± 2.40	17.20 ± 10.60	2 weeks
				15	dapagliflozin10 mg/day + Insulin	37.50 ± 15.20	8.39 ± 0.82	25.80 ± 4.80	18.10 ± 14.00	
				13	placebo + insulin	34.50 ± 12.20	8.75 ± 0.92	25.30 ± 3.00	16.20 ± 9.70	
Henry 2015-2 (23)	NCT02139943	USA	351	117	canagliflozin 100 mg/day + insulin	42.00 ± 11.60	7.90 ± 0.50	28.00 ± 3.90	22.00 ± 11.50	18 weeks
				117	canagliflozin 300 mg/day + insulin	42.80 ± 11.00	8.00 ± 0.50	28.10 ± 3.90	21.90 ± 10.60	
				117	placebo + insulin	42.00 ± 11.90	7.90 ± 0.60	28.00 ± 3.60	23.30 ± 11.00	
Kaku 2019-1 (24)	NCT02897219	Japan	174	115	ipragliflozin 50 mg/day + insulin	49.70 ± 13.10	8.68 ± 0.81	24.67 ± 2.95	NR	24 weeks
				59	placebo + insulin	48.30 ± 12.80	8.67 ± 0.79	24.21 ± 2.82		
Kaku 2019 (25)	NCT02529449	Japan	32	12	ipragliflozin 50 mg/day + insulin	43.40 ± 12.30	8.45 ± 0.76	24.54 ± 3.77	15.00 ± 9.52	2 weeks
				10	ipragliflozin 100 mg/day + insulin	41.70 ± 14.00	8.85 ± 0.72	24.25 ± 2.64	10.85 ± 5.21	
				10	placebo + insulin	44.80 ± 13.20	8.66 ± 0.74	23.93 ± 2.72	17.83 ± 8.26	
Kuhadiya 2016 (26)	NCT02518945	USA	26	17	dapagliflozin10 mg/day+insulin and liraglutide	55.00 ± 3.00	7.80 ± 0.21	31.00 ± 1.00	25.00 ± 3.00	12 weeks
				9	placebo+insulin and liraglutide	52.00 ± 3.00	7.40 ± 0.20	27.00 ± 2.00	31.00 ± 5.00	
Mathieu 2018 (27)	NCT02460978	multinational	813	271	dapagliflozin 5 mg/day+ insulin	42.70 ± 13.35	8.45 ± 0.69	27.27 ± 5.13	19.35 ± 11.79	52 weeks
				270	dapagliflozin 10 mg/day+ insulin	42.40 ± 12.80	8.43 ± 0.69	27.8 ± 5.53	19.45 ± 11.90	
				272	placebo + insulin	43.00 ± 13.73	8.43 ± 0.65	27.62 ± 5.41	18.98 ± 11.65	
Pieber 2015 (28)	NCT01969747	Austria、Germany	56	19	empagliflozin 10 mg/day + insulin	39.60 ± 11.60	8.28 ± 0.79	27.40 ± 3.50	16.20 ± 8.40	4 weeks
				18	empagliflozin 25 mg/day + insulin	41.90 ± 9.70	8.15 ± 0.54	25.40 ± 3.50	23.70 ± 14.50	
				19	placebo + insulin	40.50 ± 10.60	8.18 ± 0.67	25.40 ± 3.70	20.50 ± 12.80	
Rosenstock 2018(EASE-2) (29)	NCT02414958	multinational	723	243	empagliflozin 10 mg/day + insulin	45.70 ± 12.50	8.10 ± 0.60	29.50 ± 5.50	22.80 ± 12.60	52 weeks
				241	empagliflozin 25 mg/day + insulin	45.30 ± 13.90	8.06 ± 0.53	29.50 ± 6.00	22.50 ± 13.00	
				239	placebo + insulin	44.50 ± 13.50	8.13 ± 0.57	28.50 ± 5.30	22.40 ± 12.40	
Rosenstock 2018(EASE-3) (29)	NCT02580591	multinational	724	244	empagliflozin 10 mg/day + insulin	42.40 ± 13.30	8.19 ± 0.64	28.70 ± 5.10	20.50 ± 11.90	26 weeks
				242	empagliflozin 25 mg/day + insulin	44.20 ± 13.50	8.19 ± 0.65	28.40 ± 5.60	21.20 ± 11.40	
				238	placebo + insulin	42.20 ± 13.20	8.19 ± 0.58	27.80 ± 5.10	21.70 ± 13.00	
Sands 2015 (30)	NCT01742208	USA	33	16	sotagliflozin 400 mg/day + insulin	45.50	7.94 ± 0.55	27.10 ± 3.10	16.80	4.1 weeks
				17	placebo + insulin	34.00	7.98 ± 0.51	26.20 ± 3.00	18.50	
Shimada 2018 (31)	NCT02702011	Japan	35	12	empagliflozin 10 mg/day + insulin	44.50 ± 11.80	8.12 ± 0.37	22.68 ± 3.27	14.30 ± 8.40	4weeks
				12	empagliflozin 25 mg/day + insulin	46.60 ± 10.80	7.89 ± 0.91	22.60 ± 2.70	20.80 ± 13.50	
				11	placebo + insulin	43.90 ± 11.70	8.23 ± 0.47	23.70 ± 2.60	14.80 ± 10.00	
Watada 2018 (32)	NCT02582840	Japan	42	14	dapagliflozin 5 mg/day+ insulin	37.00 ± 10.10	7.90 ± 0.60	23.00 ± 2.30	15.90 ± 9.20	1 weeks
				14	dapagliflozin10 mg/day+ insulin	37.10 ± 10.20	7.90 ± 0.60	22.20 ± 2.10	14.70 ± 12.40	
				14	placebo+insulin	42.60 ± 10.60	8.10 ± 0.80	22.90 ± 3.40	16.90 ± 10.50	
NCT02582814	NCT02582814	Japan	151	76	dapagliflozin 5 mg/day+ insulin	47.70 ± 12.85	8.43 ± 0.71	25.28 ± 3.64	14.60 ± 8.42	52 weeks
				75	dapagliflozin 10 mg/day+ insulin	48.90 ± 12.93	8.40 ± 0.72	24.66 ± 2.81	15.94 ± 10.33	

HbA1c, Hemoglobin A1c; BMI, Body mass index.

### 3.2 Risk of bias of included studies

The overall risk of bias was some concerns. The assessment of bias risk for the included studies can be found in **Supplementary Table 4**. Among the risk of bias arising from the randomization process, one study had some concerns, while the others had a low risk. Regarding the risk of bias due to deviations from the intended interventions, 15 studies had some concerns, and the rest were at low risk. The risk of bias due to missing outcome data, risk of bias in the measurement of the outcome, and risk of bias in the selection of the reported result were all low risk. The overall assessment of the quality of the studies revealed that more than half of the studies exhibited a low risk of bias.

### 3.3 Results of network meta-analysis

**Figure 2:**
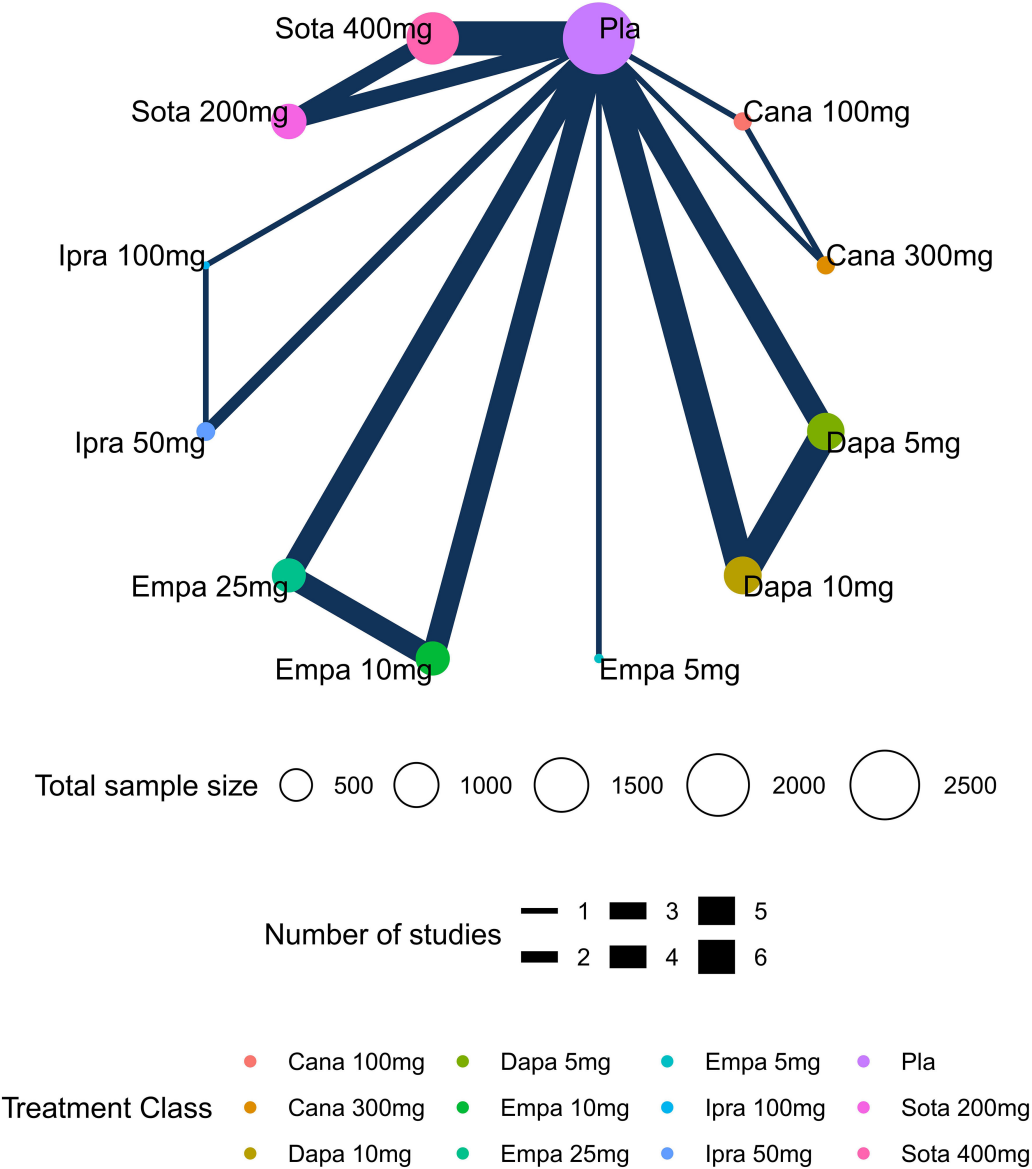
Network plots of diabetic ketoacidosis (DKA). Nodes in different colors indicate different processing. The node size corresponds to the number of participants treated in the study. The thickness of the edge represents the number of tests. The lack of lines suggests that there have been no head-to-head trials of this outcome between the two treatments.

Network plots depicting the results and quality of evidence for different doses of SGLT2 inhibitors are presented in **Figure 2**. In this diagram, each node symbolizes different doses of SGLT2 inhibitors, whereby the node’s size corresponds to the sample size associated with that intervention. Furthermore, the thickness of the lines connecting the nodes signifies the number of included studies for that specific intervention. The evaluation of inconsistency in the network meta-analysis is shown in **Supplementary Figure 1**. Additionally, heterogeneity and sensitivity of the network meta-analysis were assessed (**Supplementary Table 5**, **Supplementary Figures 2**-**4**). The results of the heterogeneity test indicated that the differences between the included studies were not statistically significant and were homogeneous (P>0.05).

### 3.4 DKA

**Figure 3:**
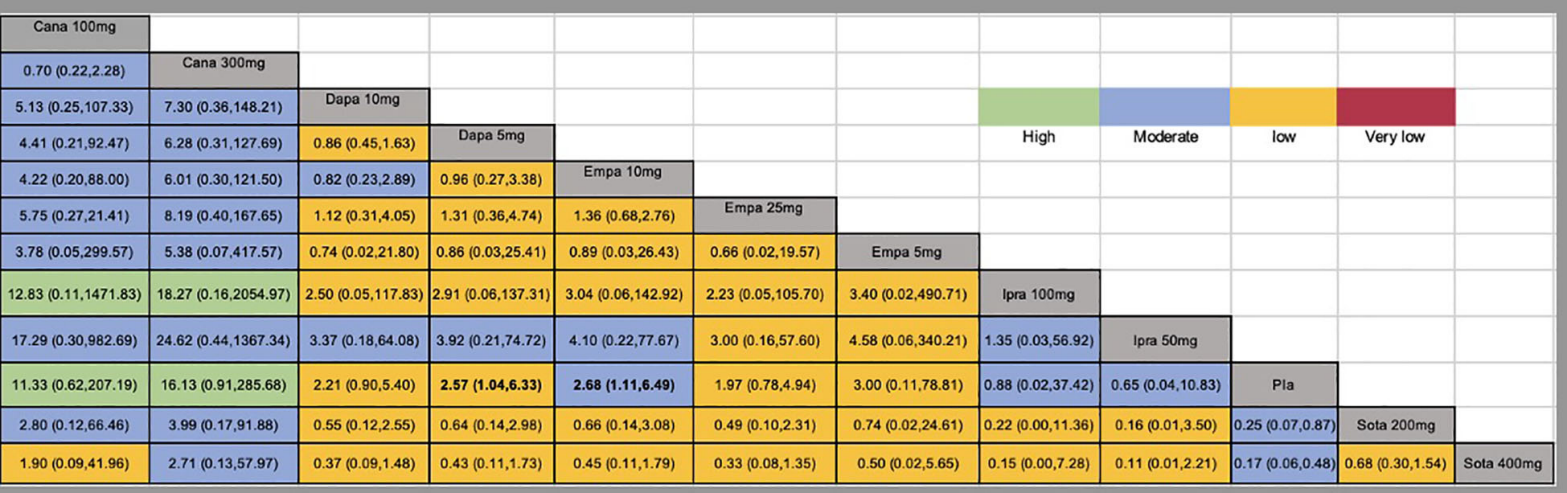
Network estimates (league tables) for different doses of SGLT2 inhibitors. Outcome: The risk of DKA (odds ratio; 95% confidence interval). The league table presented the relative effects of different kinds of SGLT2 inhibitors (the risk of DKA on the column to the risk of DKA of the row). SGLT2, Sodium-glucose co-transporter-2; Cana, Canagliflozin; Dapa, Dapagliflozin; Ipra, Ipragliflozin; Empa, Empagliflozin; Sota, sotagliflozin; Pla, Placebo; DKA, diabetic ketoacidosis.

A total of twenty studies, covering 7183 patients, reported the risk of DKA, with a total of 135 DKA events and an incidence rate of 1.88%. In this network meta-analysis, the intervention node included different doses of SGLT2 inhibitors. Compared to the placebo, dapagliflozin 5 mg,empagliflozin 10 mg, sotagliflozin 200mg and sotagliflozin 400mg had significantly greater incidences of DKA (**Figure 3**). The global *I²* for the pairwise comparison was 0%, and for the consistency model, the global *I²* was 0%. The analysis of node split indicated that the results were consistent. The GRADE quality of the network meta-analysis can be seen in **Supplementary Table 4**.

### 3.5 Rankings and P scores

The P scores of DKA for various doses of SGLT2 inhibitors are shown in **Table 2**. A high P score indicated a lower risk of DKA. The P scores of DKA suggested that different doses of SGLT2 inhibitors were associated with different risks of DKA. **Table 2** shows that canagliflozin 300 mg had the lowest risk of DKA (P score = 0.8563).

**Table 2:** Frequentist P-score for different doses of SGLT2 inhibitors.

The risk of DKA (odds ratio; 95% confidence interval)		
Intervention	Intervention vs Pla	Frequentist P-score
Cana 300mg	16.13 (0.91, 285.68)	0.8563
Cana 100mg	11.33 (0.62, 207.19)	0.7724
Sota 400mg	5.96 (2.06, 17.20)	0.7679
Sota 200mg	4.04 (1.15, 14.18)	0.6226
Empa 5mg	3.00 (0.11, 78.81)	0.5178
Empa 10mg	2.68 (1.11, 6.49)	0.5166
Dapa 5mg	0.99 (0.02,50.39)	0.4967
Dapa 10mg	2.21 (0.90, 5.40)	0.4250
Empa 25mg	1.97 (0.78, 4.94)	0.3801
Ipra 100mg	0.88 (0.02, 37.42)	0.2950
Ipra 50mg	0.65 (0.04, 10.83)	0.2003
Pla	–	0.1493

Cana, Canagliflozin; Dapa, Dapagliflozin; Ipra, Ipragliflozin; Empa, Empagliflozin; Sota, Sotagliflozin; Pla, Placebo; DKA, Diabetic ketoacidosis.

### 3.6 Funnel plot and sensitivity analysis

Egger’s test indicated that there was no publication bias among the different doses of SGLT2 inhibitors (*p* = 0.72, **Supplementary Figure 5**). **Supplementary Table 5** shows the results of the sensitivity analyses. The results showed that all sensitivity analyses were consistent with the primary results.

Sensitivity analysis using the Bayesian framework revealed that 100 mg canagliflozin and 300 mg canagliflozin were associated with a greater risk of DKA. Additionally, 10 mg empagliflozin had a greater risk than 25 mg empagliflozin. Other sensitivity analyses were consistent with the primary results.

## 4 Discussion

At present, SGLT2 inhibitors are exclusively approved for the treatment of T2DM. However, there are potential advantages to utilizing SGLT2 inhibitors in the adjuvant treatment of T1DM, such as better blood sugar control, decreased insulin dosage, weight management and loss, lower blood pressure, and improved blood sugar fluctuation (34). Although there have been reports of SGLT-2i being used for type 1 diabetes, it remains unclear whether such off-label drug use poses any safety risks. According to studies, SGLT-2i might increase the incidence of DKA in T1DM patients, which is a rare occurrence in T2DM. We conducted relevant research during the previous period and discovered that SGLT2 inhibitors were not associated with an elevated risk of DKA compared to the placebo in the treatment of type 2 diabetes. Furthermore, no dose-dependent association was observed between SGLT2 inhibitors and the risk of DKA in type 2 diabetes (35). According to the results of the network meta-analysis, SGLT2 inhibitors were not associated with a greater risk of DKA than was a placebo. Except for dapagliflozin 5 mg,empagliflozin 10 mg, sotagliflozin 200mg and sotagliflozin 400mg no risk of DKA was observed with SGLT2 inhibitors as an adjunct to insulin in the treatment of T1DM compared to insulin.

A study has been conducted to compare the safety and efficacy of different hypoglycemic agents when used as adjunctive therapy for T1DM. It was concluded that the risk of DKA and the risk of genital infection were more common with SGLT2 inhibitors than with other hypoglycemic agents (36). However, the study did not compare the incidence of DKA between different doses of SGLT inhibitors. This research is a network meta-analysis of SGLT2 inhibitors at different doses for the treatment of T1DM. The results showed that dapagliflozin 5 mg,empagliflozin 10 mg, sotagliflozin 200mg and sotagliflozin 400mg for the treatment of T1DM had a significantly greater incidence of DKA than the placebo. According to the P scores, SGLT2 inhibitors were associated with a lower risk of DKA than was a placebo, and 300 mg canagliflozin had the lowest risk of DKA, but this difference was not statistically significant compared to that associated with a placebo.

There have also been some reports of DKA caused by dapagliflozin, but the most common primary causes of DKA are insulin pump failure and a missed insulin dose (22). Most of the studies that started adjuvant avoidance of excessive insulin dose reductions at the time of dapagliflozin therapy (18, 26, 28). Subsequent careful reduction of the insulin dose during therapy may be important for reducing the risk of DKA. For empagliflozin, the incidence of DKA was dose-related, and the risk of DKA was associated with concomitant disease or infection with excessive insulin dose reductions or insulin pump failure. The likelihood of DKA was similar for 10 mg and 25 mg, but the clinical manifestations were more severe with 25 mg (29). The study concluded that the incidence of DKA was greater in female patients and those who used insulin pumps. If an SGLT inhibitor is considered for T1DM patients, it should not be used with a low-carbohydrate diet or in patients with a history of excessive alcohol consumption or recent episodes of DKA. Regarding sotagliflozin, six of the studies we included were of sotagliflozin therapy adjunctive to the treatment T1DM (15–17, 19, 20, 30); the overall results suggest that sotagliflozin 400 mg is more susceptible to DKA. after the occurrence of DKA, the majority of patients recovered from discontinuation of sotagliflozin, whereas it is thought that one of the causes of DKA is still the inappropriate operation of or malfunctioning in the use of the insulin pumps. Therefore, it is considered necessary to monitor ketosis while using sotagliflozin and to consider discontinuing the drug before scheduled surgical procedures (17).

In 2020, the National NICE guidance identified dapagliflozin as a treatment with both clinical and economic value for people with T1DM. Furthermore, the guidance provides recommendations for when prescribing dapagliflozin would be appropriate in patients with T1DM. However, in 2021, dapagliflozin combined with insulin was no longer licensed for treating T1DM. Dapagliflozin and sotagliflozin were previously approved for the treatment of T1DM in Europe but were never approved in the United States due to the lack of adequate data on the increased risk of DKA reported in clinical trials, including episodes of euglycemic DKA. Currently, the latest UK Kidney Association guidelines recommend the use of SGLT2 inhibitors in patients with T1DM if they have an eGFR ≥ 20 mL/min/1.73 m2 and a uACR ≥ 25 mg/mmol under the strict direction of a specialized diabetologist (37).

SGLT2 inhibitors can significantly lower glucose levels by reducing glucose reabsorption from the kidneys, resulting in more glucose being excreted through the urine. One of the advantages of SGLT2 inhibitors is that they operate independently of insulin secretion, which means that their efficacy remains unaffected by insulin resistance or β-cell dysfunction. Therefore, the non-insulin-dependent mode of action of SGLT2 inhibitors can also benefit patients with T1DM. Adjuvant therapy with SGLT2 inhibitors reduces the total daily insulin dose in patients with T1DM, and ketone bodies increase when low-dose insulin is insufficient to inhibit lipolysis in peripheral adipose tissue. Therefore, the use of SGLT2 inhibitors in patients with T1DM may increase the incidence of ketone body-related events. However, a significant increase in real-world research evidence is needed to guide clinicians in assessing the true risk of DKA in patients with T1DM under the rigorous scrutiny of clinical trials through careful patient selection and intensive patient and clinical team education.

### 4.1 Strengths and limitations

This study is the first network meta-analysis of SGLT2 inhibitors in patients with T1DM investigating the risk of DKA. In addition, we included one unpublished trial from a clinical trial database that provided additional DKA data. Previously, we also conducted a systematic review and network meta-analysis of the available evidence for the risk of DKA associated with SGLT2 inhibitors in patients with T2DM (36). Several potential limitations should be acknowledged. First, there are few clinical studies on the use of SGLT2 inhibitors in the treatment of T1DM, and the incidence of DKA is low, which may be biased. Second, none of the included studies distinguished eDKA from DKA. Therefore, our outcome was DKA, not eDKA.

## 5 Conclusion

Except for 5mg dapagliflozin,10mg empagliflozin,200mg sotagliflozin and 400mg sotagliflozin, no risk of DKA was observed with SGLT2 inhibitors as an adjunct to insulin in the treatment of T1DM compared to insulin. Considering the glucose-lowering mechanism of SGLT2 inhibitors, SGLT2 inhibitors may be useful as an adjunct to insulin for treating T1DM. If available, we recommend canagliflozin because of the P score. However, the treatment of T1DM with SGLT2 inhibitors is off-label, and adverse reactions should be closely monitored during administration.

## Data Availability

The original contributions presented in the study are included in the article/**Supplementary Material**. Further inquiries can be directed to the corresponding author.
