# Supplementary material for: Risk of diabetic ketoacidosis caused by sodium glucose cotransporter-2 inhibitors in patients with type 1 diabetes: a systematic review and network meta-analysis of randomized controlled trials
Source: Front Endocrinol (Lausanne). 2025 Jan 31;15:1453067. doi: 10.3389/fendo.2024.1453067 (PMC11826237; doi:10.3389/fendo.2024.1453067)

***Supplementary Material***

Risk of diabetic ketoacidosis caused by sodium glucose cotransporter-2 inhibitors in patients with type 1 diabetes: a systematic review and network meta-analysis of randomized controlled trials.

Ying Liu, Shiwen Yang, Aidou Jiang, Dan Zou, Zhaoyang Chen, Na Su^*^

*** Correspondence:** Na Su: [zoya159@163.com](mailto:zoya159@163.com).

## Supplementary Table 1. PRISMA Checklist. PRISMA checklist for this network meta-analysis

| **Section/topic** | **#** | **Checklist item** | **Reported on page #** |
| --- | --- | --- | --- |
| **TITLE** | | |  |
| Title | 1 | Identify the report as a systematic review incorporating a network meta-analysis (or related form of meta-analysis). | 1 |
| **ABSTRACT** | | |  |
| Structured summary | 2 | Provide a structured summary including, as applicable: Background: main objectives  Methods: data sources; study eligibility criteria, participants, and interventions; study appraisal; and synthesis methods, such as network meta-analysis.  Results: number of studies and participants identified; summary estimates with corresponding confidence/credible intervals; treatment rankings may also be discussed. Authors may choose to summarize pairwise comparisons against a chosen treatment included in their analyses for brevity.  Discussion/Conclusions: limitations; conclusions and implications of findings.  Other: primary source of funding; systematic review registration number with registry name. | 2, 3 |
| **INTRODUCTION** | | |  |
| Rationale | 3 | Describe the rationale for the review in the context of what is already known, including mention of why a network meta-analysis has been conducted. | 4,5 |
| Objectives | 4 | Provide an explicit statement of questions being addressed with reference to participants, interventions, comparisons, outcomes, and study design (PICOS). | 4,5 |
| **METHODS** | | |  |
| Protocol and registration | 5 | Indicate if a review protocol exists, if and where it can be accessed (e.g., Web address), and, if available, provide registration information including registration number. | 5 |
| Eligibility criteria | 6 | Specify study characteristics (e.g., PICOS, length of follow-up) and report characteristics (e.g., years considered, language, publication status) used as criteria for eligibility, giving rationale.  Clearly describe eligible treatments included in the treatment network, and note whether any have been clustered or merged into the same node (with justification). | 5,6 |
| Information sources | 7 | Describe all information sources (e.g., databases with dates of coverage, contact with study authors to identify additional studies) in the search and date last searched. | 5, 6 |
| Search | 8 | Present full electronic search strategy for at least one database, including any limits used, such that it could be repeated. | 5, 6 |
| Study selection | 9 | State the process for selecting studies (i.e., screening, eligibility, included in systematic review, and, if applicable, included in the meta-analysis). | 5, 6 |
| Data collection process | 10 | Describe method of data extraction from reports (e.g., piloted forms, independently, in duplicate) and any processes for obtaining and confirming data from investigators. | 6 |
| Data items | 11 | List and define all variables for which data were sought (e.g., PICOS, funding sources) and any assumptions and simplifications made. | 6 |
| Risk of bias in individual studies | 12 | Describe methods used for assessing risk of bias of individual studies (including specification of whether this was done at the study or outcome level), and how this information is to be used in any data synthesis. | 6 |
| Summary measures | 13 | State the principal summary measures (e.g., risk ratio, difference in means). Also describe the use of additional summary measures assessed, such as treatment rankings and surface under the cumulative ranking curve (SUCRA) values, as well as modified approaches used to present summary findings from meta-analyses. | 6 |
| Planned methods of analysis | 14 | Describe the methods of handling data and combining results of studies for each network meta-analysis. This should include, but not be limited to:  Handling of multigroup trials;  Selection of variance structure;  Selection of prior distributions in Bayesian analyses; and  Assessment of model fit. | 6,7 |
| Assessment of inconsistency | S2 | Describe the statistical methods used to evaluate the agreement of direct and indirect evidence in the treatment network(s) studied. Describe efforts taken to address its presence when found. | 7 |
| Risk of bias across studies | 15 | Specify any assessment of risk of bias that may affect the cumulative evidence (e.g., publication bias, selective reporting within studies). | 7 |
| Additional analyses | 16 | Describe methods of additional analyses if done, indicating which were prespecified. This may include, but not be limited to, the following:  Sensitivity or subgroup analyses;  Meta-regression analyses;  Alternative formulations of the treatment network; and  Use of alternative prior distributions for Bayesian analyses (if applicable). | 7 |
| **RESULTS** |  |  |  |
| Study selection | 17 | Give numbers of studies screened, assessed for eligibility, and included in the review, with reasons for exclusions at each stage, ideally with a flow diagram. | 7, Figure 1 |
| Presentation of network structure | S3 | Provide a network graph of the included studies to enable visualization of the geometry of the treatment network. | Figure 1 |
| Summary of network geometry | S4 | Provide a brief overview of characteristics of the treatment network. This may include commentary on the abundance of trials and randomized patients for the different interventions and pairwise comparisons in the network, gaps of evidence in the treatment network, and potential biases reflected by the network structure. | 8,9 |
| Study characteristics | 18 | For each study, present characteristics for which data were extracted (e.g., study size, PICOS, follow-up period) and provide the citations. | 7, 8  Table 1 |
| Risk of bias within studies | 19 | Present data on risk of bias of each study and, if available, any outcome level assessment. | 8 |
| Results of individual studies | 20 | For all outcomes considered (benefits or harms), present, for each study: 1) simple summary data for each intervention group, and 2) effect estimates and confidence intervals. Modified approaches may be needed to deal with information from larger networks. | 9 |
| Synthesis of results | 21 | Present results of each meta-analysis done, including confidence/credible intervals. In larger networks, authors may focus on comparisons versus a particular comparator (e.g., placebo or standard care), with full findings presented in an appendix. League tables and forest plots may be considered to summarize pairwise comparisons. If additional summary measures were explored (such as treatment rankings), these should also be presented. | 9, 10  Figure 2  Figure 3 |
| Exploration for inconsistency | S5 | Describe results from investigations of inconsistency. This may include such information as measures of model fit to compare consistency and inconsistency models, P values from statistical tests, or summary of inconsistency estimates from different parts of the treatment network. | 9, 10 |
| Risk of bias across studies | 22 | Present results of any assessment of risk of bias across studies (see Item 15). | 8 |
| Additional analysis | 23 | Give results of additional analyses, if done (e.g., sensitivity or subgroup analyses, meta-regression analyses, alternative network geometries studied, alternative choice of prior distributions for Bayesian analyses, and so forth). | 9,10 |
| **DISCUSSION** |  |  |  |
| Summary of evidence | 24 | Summarize the main findings including the strength of evidence for each main outcome; consider their relevance to key groups (e.g., healthcare providers, users, and policy makers). | 10, 11, 12 |
| Limitations | 25 | Discuss limitations at study and outcome level (e.g., risk of bias), and at review-level (e.g., incomplete retrieval of identified research, reporting bias). | 13 |
| Conclusions | 26 | Provide a general interpretation of the results in the context of other evidence, and implications for future research. | 13 |
| **FUNDING** |  |  |  |
| Funding | 27 | Describe sources of funding for the systematic review and other support (e.g., supply of data); role of funders for the systematic review. | 1 |

**Supplementary Table 2. PRISMA NMA Checklist of Items to Include When Reporting A Systematic Review Involving a Network Meta-analysis**

| **Section/Topic** | **Item #** | **Checklist Item** | **Reported on Page #** |
| --- | --- | --- | --- |
| **TITLE** |  |  |  |
| Title | 1 | Identify the report as a systematic review *incorporating a network meta-analysis (or related form of meta-analysis).* | 1 |
|  |  |  |  |
| **ABSTRACT** |  |  |  |
| Structured summary | 2 | Provide a structured summary including, as applicable:  **Background:** main objectives  **Methods:** data sources; study eligibility criteria, participants, and interventions; study appraisal; and *synthesis methods, such as network meta-analysis.*  **Results:** number of studies and participants identified; summary estimates with corresponding confidence/credible intervals; *treatment rankings may also be discussed. Authors may choose to summarize pairwise comparisons against a chosen treatment included in their analyses for brevity.*  **Discussion/Conclusions:** limitations; conclusions and implications of findings.  **Other:** primary source of funding; systematic review registration number with registry name. | 2 |
|  |  |  |  |
| **INTRODUCTION** |  |  |  |
| Rationale | 3 | Describe the rationale for the review in the context of what is already known*, including mention of why a network meta-analysis has been conducted.* | 4 |
| Objectives | 4 | Provide an explicit statement of questions being addressed, with reference to participants, interventions, comparisons, outcomes, and study design (PICOS). | 5 |
|  |  |  |  |
| **METHODS** |  |  |  |
| Protocol and registration | 5 | Indicate whether a review protocol exists and if and where it can be accessed (e.g., Web address); and, if available, provide registration information, including registration number. | 5 |
| Eligibility criteria | 6 | Specify study characteristics (e.g., PICOS, length of follow-up) and report characteristics (e.g., years considered, language, publication status) used as criteria for eligibility, giving rationale. *Clearly describe eligible treatments included in the treatment network, and note whether any have been clustered or merged into the same node (with justification).* | 6 |
| Information sources | 7 | Describe all information sources (e.g., databases with dates of coverage, contact with study authors to identify additional studies) in the search and date last searched. | 5 |
| Search | 8 | Present full electronic search strategy for at least one database, including any limits used, such that it could be repeated. | 5 |
| Study selection | 9 | State the process for selecting studies (i.e., screening, eligibility, included in systematic review, and, if applicable, included in the meta-analysis). | 6 |
| Data collection process | 10 | Describe method of data extraction from reports (e.g., piloted forms, independently, in duplicate) and any processes for obtaining and confirming data from investigators. | 6 |
| Data items | 11 | List and define all variables for which data were sought (e.g., PICOS, funding sources) and any assumptions and simplifications made. | 6 |
| **Geometry of the network** | **S1** | Describe methods used to explore the geometry of the treatment network under study and potential biases related to it. This should include how the evidence base has been graphically summarized for presentation, and what characteristics were compiled and used to describe the evidence base to readers. | 6,7 |
| Risk of bias within individual studies | 12 | Describe methods used for assessing risk of bias of individual studies (including specification of whether this was done at the study or outcome level), and how this information is to be used in any data synthesis. | 6 |
| Summary measures | 13 | State the principal summary measures (e.g., risk ratio, difference in means). *Also describe the use of additional summary measures assessed, such as treatment rankings and surface under the cumulative ranking curve (SUCRA) values, as well as modified approaches used to present summary findings from meta-analyses.* | 6,7 |
| Planned methods of analysis | 14 | Describe the methods of handling data and combining results of studies for each network meta-analysis. This should include, but not be limited to:   - *Handling of multi-arm trials;* - *Selection of variance structure;* - *Selection of prior distributions in Bayesian analyses; and* - *Assessment of model fit.* | 6,7 |
| **Assessment of Inconsistency** | **S2** | Describe the statistical methods used to evaluate the agreement of direct and indirect evidence in the treatment network(s) studied. Describe efforts taken to address its presence when found. | 6,7 |
| Risk of bias across studies | 15 | Specify any assessment of risk of bias that may affect the cumulative evidence (e.g., publication bias, selective reporting within studies). | 8 |
| Additional analyses | 16 | Describe methods of additional analyses if done, indicating which were pre-specified. This may include, but not be limited to, the following:   - Sensitivity or subgroup analyses; - Meta-regression analyses; - *Alternative formulations of the treatment network; and* - *Use of alternative prior distributions for Bayesian analyses (if applicable).* | 8 |
|  |  |  |  |
| **RESULTS†** |  |  |  |
| Study selection | 17 | Give numbers of studies screened, assessed for eligibility, and included in the review, with reasons for exclusions at each stage, ideally with a flow diagram. | 7,8 |
| **Presentation of network structure** | **S3** | Provide a network graph of the included studies to enable visualization of the geometry of the treatment network. | 8 |
| **Summary of network geometry** | **S4** | Provide a brief overview of characteristics of the treatment network. This may include commentary on the abundance of trials and randomized patients for the different interventions and pairwise comparisons in the network, gaps of evidence in the treatment network, and potential biases reflected by the network structure. | 8,9 |
| Study characteristics | 18 | For each study, present characteristics for which data were extracted (e.g., study size, PICOS, follow-up period) and provide the citations. | 8,9 |
| Risk of bias within studies | 19 | Present data on risk of bias of each study and, if available, any outcome level assessment. | 8 |
| Results of individual studies | 20 | For all outcomes considered (benefits or harms), present, for each study: 1) simple summary data for each intervention group, and 2) effect estimates and confidence intervals. *Modified approaches may be needed to deal with information from larger networks.* | 9 |
| Synthesis of results | 21 | Present results of each meta-analysis done, including confidence/credible intervals. *In larger networks, authors may focus on comparisons versus a particular comparator (e.g. placebo or standard care), with full findings presented in an appendix. League tables and forest plots may be considered to summarize pairwise comparisons.* If additional summary measures were explored (such as treatment rankings), these should also be presented. | 9 |
| **Exploration for inconsistency** | **S5** | Describe results from investigations of inconsistency. This may include such information as measures of model fit to compare consistency and inconsistency models, *P* values from statistical tests, or summary of inconsistency estimates from different parts of the treatment network. | 9 |
| Risk of bias across studies | 22 | Present results of any assessment of risk of bias across studies for the evidence base being studied. | 8 |
| Results of additional analyses | 23 | Give results of additional analyses, if done (e.g., sensitivity or subgroup analyses, meta-regression analyses*, alternative network geometries studied, alternative choice of prior distributions for Bayesian analyses,* and so forth). | 10 |
|  |  |  |  |
| **DISCUSSION** |  |  |  |
| Summary of evidence | 24 | Summarize the main findings, including the strength of evidence for each main outcome; consider their relevance to key groups (e.g., healthcare providers, users, and policy-makers). | 10,11,12 |
| Limitations | 25 | Discuss limitations at study and outcome level (e.g., risk of bias), and at review level (e.g., incomplete retrieval of identified research, reporting bias). *Comment on the validity of the assumptions, such as transitivity and consistency. Comment on any concerns regarding network geometry (e.g., avoidance of certain comparisons).* | 13 |
| Conclusions | 26 | Provide a general interpretation of the results in the context of other evidence, and implications for future research. | 13 |
|  |  |  |  |
| **FUNDING** |  |  |  |
| Funding | 27 | Describe sources of funding for the systematic review and other support (e.g., supply of data); role of funders for the systematic review. This should also include information regarding whether funding has been received from manufacturers of treatments in the network and/or whether some of the authors are content experts with professional conflicts of interest that could affect use of treatments in the network. | 14 |

**Supplementary Table 3. Search strategy.**

| **Database** | **Search strategy** |
| --- | --- |
| PubMed | Search: ((((sglt [tw]) OR ((sglt2 [tw]) OR (Sodium-Glucose Transport Proteins [mh]))) OR ((((((((((((((((((((((((((((((gliflozins[mh]) OR (gliflozins[tw])) OR (bexagliflozin[mh])) OR (bexagliflozin[tw])) OR (canagliflozin[mh])) OR (canagliflozin[tw])) OR (dapagliflozin[mh])) OR (dapagliflozin[tw])) OR (empagliflozin[mh])) OR (empagliflozin[tw])) OR (ertugliflozin[mh])) OR (ertugliflozin[tw])) OR (henagliflozin[mh])) OR (henagliflozin[tw])) OR (ipragliflozin[mh])) OR (ipragliflozin[tw])) OR (licogliflozin[mh])) OR (licogliflozin[tw])) OR (luseogliflozin[mh])) OR (luseogliflozin[tw])) OR (remogliflozins[mh])) OR (remogliflozins[tw])) OR (sergliflozin[mh])) OR (sergliflozin[tw])) OR (sotagliflozin[mh])) OR (sotagliflozin[tw])) OR (tofogliflozin[mh])) OR (tofogliflozin[tw])) OR (gliflozins[mh])) OR (gliflozins[tw]))) AND (Diabetes Mellitus, Type 1[mh])) AND (randomized controlled trial[pt] OR controlled clinical trial[pt] OR clinical trials as topic [mesh: noexp] OR randomized[tiab] OR randomised [tiab] OR placebo [tiab] OR randomly [tiab] OR trial [ti] OR drug therapy [sh] OR groups [tiab])  Search: ((ketoacidosis[tw] OR ketoacidosis[mh])) AND (((((sglt [tw]) OR ((sglt2 [tw]) OR (Sodium-Glucose Transport Proteins [mh]))) OR ((((((((((((((((((((((((((((((gliflozins[mh]) OR (gliflozins[tw])) OR (bexagliflozin[mh])) OR (bexagliflozin[tw])) OR (canagliflozin[mh])) OR (canagliflozin[tw])) OR (dapagliflozin[mh])) OR (dapagliflozin[tw])) OR (empagliflozin[mh])) OR (empagliflozin[tw])) OR (ertugliflozin[mh])) OR (ertugliflozin[tw])) OR (henagliflozin[mh])) OR (henagliflozin[tw])) OR (ipragliflozin[mh])) OR (ipragliflozin[tw])) OR (licogliflozin[mh])) OR (licogliflozin[tw])) OR (luseogliflozin[mh])) OR (luseogliflozin[tw])) OR (remogliflozins[mh])) OR (remogliflozins[tw])) OR (sergliflozin[mh])) OR (sergliflozin[tw])) OR (sotagliflozin[mh])) OR (sotagliflozin[tw])) OR (tofogliflozin[mh])) OR (tofogliflozin[tw])) OR (gliflozins[mh])) OR (gliflozins[tw]))) AND (Diabetes Mellitus, Type 1 [mh])) AND (randomized controlled trial[pt] OR controlled clinical trial[pt] OR clinical trials as topic [mesh: noexp] OR randomized[tiab] OR randomised [tiab] OR placebo [tiab] OR randomly [tiab] OR trial [ti] OR drug therapy [sh] OR groups [tiab])) |
| Embase(via OVID) | (exp insulin dependent diabetes mellitus/ OR (Diabetes Mellitus, Type 1 or Type 1 Diabetes Mellitus or iddm* or DMT1 or T1DM).mp.) AND (exp sodium glucose cotransporter 2/ or exp sodium glucose cotransporter 2 inhibitor/ or sglt.mp. or sglt-2.mp. or sglt2.mp. or Sodium-Glucose Transport Protein$.mp. or Sodium-glucose transporter$.mp. or Sodium-glucose co-transporter$.mp. or Sodium glucose cotransporter$.mp. or gliflozins.mp. or exp gliflozins/ or bexagliflozin.mp. or exp bexagliflozin/ or canagliflozin.mp. or exp canagliflozin/ or dapagliflozin.mp. or exp dapagliflozin/ or empagliflozin.mp. or exp empagliflozin/ or ertugliflozin.mp. or exp ertugliflozin/ or henagliflozin.mp. or exp henagliflozin/ or ipragliflozin.mp. or exp ipragliflozin/ or licogliflozin.mp. or exp licogliflozin/ or luseogliflozin.mp. or exp luseogliflozin/ or remogliflozin.mp. or exp remogliflozin etabonate/ or sergliflozin.mp. or exp sergliflozin etabonate/ or sotagliflozin.mp. or exp sotagliflozin/ or tofogliflozin.mp. or exp tofogliflozin/ or gliflozin.mp. or exp gliflozin/) AND (Clinical trial/ or Randomized controlled trial/ or Randomization/ or Randomi?ed controlled trial$.tw. or Rct.tw. or Random allocation.tw. or Randomly allocated.tw. or Allocated randomly.tw. or (allocated adj2 random).tw.) AND (exp ketoacidosis/ or ketoacidosis.mp.) |
| Cochrane Central Register of Controlled Trials(via OVID) | (exp Diabetes Mellitus, Type 1/ OR (Diabetes Mellitus, Type 1 or Type 1 Diabetes Mellitus or iddm* or DMT1 or T1DM).mp.) AND (exp sodium glucose cotransporter 2/ or exp sodium glucose cotransporter 2 inhibitor/ or sglt.mp. or sglt-2.mp. or sglt2.mp. or Sodium-Glucose Transport Protein$.mp. or Sodium-glucose transporter$.mp. or Sodium-glucose co-transporter$.mp. or Sodium glucose cotransporter$.mp. or gliflozins.mp. or exp gliflozins/ or bexagliflozin.mp. or exp bexagliflozin/ or canagliflozin.mp. or exp canagliflozin/ or dapagliflozin.mp. or exp dapagliflozin/ or empagliflozin.mp. or exp empagliflozin/ or ertugliflozin.mp. or exp ertugliflozin/ or henagliflozin.mp. or exp henagliflozin/ or ipragliflozin.mp. or exp ipragliflozin/ or licogliflozin.mp. or exp licogliflozin/ or luseogliflozin.mp. or exp luseogliflozin/ or remogliflozin.mp. or exp remogliflozin etabonate/ or sergliflozin.mp. or exp sergliflozin etabonate/ or sotagliflozin.mp. or exp sotagliflozin/ or tofogliflozin.mp. or exp tofogliflozin/ or gliflozin.mp. or exp gliflozin/) AND (Clinical trial/ or Randomized controlled trial/ or Randomization/ or Randomi?ed controlled trial$.tw. or Rct.tw. or Random allocation.tw. or Randomly allocated.tw. or Allocated randomly.tw. or (allocated adj2 random).tw.) |

## Supplementary Table 4. Risk of bias assessments

| **Study** | **D1** | **D2** | **D3** | **D4** | **D5** | **Overall** |
| --- | --- | --- | --- | --- | --- | --- |
| Baker 2019 | Low | Some concerns | Low | Low | Low | Some concerns |
| Bode 2021 | Low | Some concerns | Low | Low | Low | Some concerns |
| Buse 2018 | Low | Some concerns | Low | Low | Low | Some concerns |
| Dandona 2018 | Low | Some concerns | Low | Low | Low | Some concerns |
| Danne 2018 | Low | Some concerns | Low | Low | Low | Some concerns |
| Garg 2017 | Low | Some concerns | Low | Low | Low | Some concerns |
| Garcia-Tirado 2022 | Low | Low | Low | Low | Low | Low |
| Henry-1 2015 | Low | Low | Low | Low | Low | Low |
| Henry-2 2015 | Low | Low | Low | Low | Low | Low |
| Kaku-1 2019 | Low | Some concerns | Low | Low | Low | Some concerns |
| Kaku-2 2019 | Low | Low | Low | Low | Low | Low |
| Kuhadiya 2016 | Low | Some concerns | Low | Low | Low | Some concerns |
| Mathieu 2020 | Low | Some concerns | Low | Low | Low | Some concerns |
| Pieber 2015 | Low | Some concerns | Low | Low | Low | Some concerns |
| Rosenstock 2018(EASE-2) | Low | Some concerns | Low | Low | Low | Some concerns |
| Rosenstock 2018(EASE-3) | Low | Some concerns | Low | Low | Low | Some concerns |
| Sands 2015 | Low | Some concerns | Low | Low | Low | Some concerns |
| Shimada 2018 | Low | Low | Low | Low | Low | Low |
| Watada 2018 | Low | Some concerns | Low | Low | Low | Some concerns |
| NCT02582814 | Some concerns | Some concerns | Low | Low | Low | Some concerns |

**Footnote:** D1: Risk of bias arising from the randomization process; D2: Risk of bias due to deviations from the intended interventions; D3: Risk of bias due to missing outcome data; D4: Risk of bias in measurement of the outcome; D5: Risk of bias in selection of the reported result; Overall: Overall risk of bias

## Supplementary Table 5. Heterogeneity assessments for different doses of SGLT-2i

| **Outcomes** | **Design-based Q statistic, comparisons, and overall statement** | **Q statistic** | **Degree of freedom** | **P value** |
| --- | --- | --- | --- | --- |
| **The risk of DKA** | **Design-specific decomposition of within-designs Q statistic** | | | |
|  | Pla vs Sota 400mg | 3.38 | 2 | 0.1841 |
|  | Pla vs Dapa 10mg vs Dapa 5mg | 2.16 | 6 | 0.9049 |
|  | Pla vs Empa 10mg vs Empa 25mg | 0.70 | 6 | 0.9945 |
|  | Pla vs Sota 200mg vs Sota 400mg | 2.51 | 4 | 0.6425 |
|  | **Between-designs Q statistic after detaching of single designs** | | | |
|  | Dapa 10mg vs Dapa 5mg | 0.36 | 3 | 0.9491 |
|  | Pla vs Dapa 10mg | 0.52 | 3 | 0.9144 |
|  | Pla vs Ipra 50mg | 0.54 | 3 | 0.9108 |
|  | Pla vs Sota 400mg | 0.28 | 3 | 0.9641 |
|  | Pla vs Dapa 10mg vs Dapa 5mg | 0.32 | 2 | 0.8537 |
|  | Q statistic to assess consistency under the assumption of a full design-by-treatment interaction random effects model: between designs Q statistic, 0.57; degree of freedom, 4; p value, 0.9668; tau.within, 0; tau2.within, 0. | | | |

**Footnote:** Q statistic: Cochran’s Q; tau.within: tau statistic within design; Dapa: Dapagliflozin; Ipra: Ipragliflozin; Empa: Empagliflozin; Sota: Sotagliflozin; Pla:Placebo; DKA: diabetic ketoacidosis.

## Supplementary Table 6. GRADE assessments for different doses of SGLT-2i

| **Comparison** | **Direct estimate** | **Certainty** | **Indirect estimate** | **Certainty** | **Network estimate** | **Certainty** |
| --- | --- | --- | --- | --- | --- | --- |
| Cana 100mg: Cana 300mg | 0.70 (0.22, 2.28) | High | - | - | 0.70 (0.22, 2.28) | Moderate^5^ |
| Cana 100mg: Pla | 11.33 (0.62, 207.19) | High | - | - | 11.33 (0.62, 207.19) | High^7^ |
| Cana 300mg: Pla | 16.13 (0.91, 285.68) | High | - | - | 16.13 (0.91, 285.68) | High^7^ |
| Dapa 10mg: Dapa 5mg | 0.86 (0.45, 1.63) | Moderate^1^ | 1.37 (0.00, 710.22) | Moderate^3^ | 0.86 (0.45, 1.63) | Low^5^ |
| Dapa 10mg: Pla | 2.36 (0.94, 5.89) | Moderate^1^ | 0.58 (0.01, 35.98) | Moderate^3^ | 2.21 (0.90, 5.40) | Low^5^ |
| Dapa 5mg: Pla | 2.53 (0.98, 6.52) | Moderate^1^ | 2.95 (0.15, 58.02) | Moderate^3^ | 2.57 (1.04, 6.33) | Low^5^ |
| Empa 10mg: Empa 25mg | 1.36 (0.67, 2.74) | Moderate^1^ | 595.54 (0.00, 24555195654533.46) | - | 1.36 (0.68, 2.76) | Low^5^ |
| Empa 10mg: Pla | 2.70 (1.11, 6.55) | Moderate^1^ | 1.18 (0.00, 37640.53) | - | 2.68 (1.11, 6.49) | Moderate^1^ |
| Empa 25mg: Pla | 2.00 (0.79, 5.02) | Moderate^1^ | 0.16 (0.00, 44493.01) | - | 1.97 (0.78, 4.94) | Low^5^ |
| Empa 5mg: Pla | 3.00 (0.11, 78.81) | Moderate^1^ | - | - | 3.00 (0.11, 78.81) | Moderate^7^ |
| Ipra 100mg: Ipra 50mg | 1.19 (0.02, 65.32) | High | 3.18(0.00, 118961.74) | Moderate^2^ | 1.35 (0.03, 56.92) | Moderate^5^ |
| Ipra 100mg: Pla | 1.00 (0.02, 55.27) | High | 0.38(0.00, 13440.98) | Moderate^2^ | 0.88 (0.02, 37.42) | Moderate^5^ |
| Ipra 50mg: Pla | 0.65 (0.04, 10.83) | Moderate^1^ | - | High^4^ | 0.65 (0.04, 10.83) | Moderate^5^ |
| Sota 200mg: Pla | 4.25 (0.81, 22.43) | Moderate^1^ | 3.77 (0.56, 25.59) | Moderate^3^ | 4.04 (1.15, 14.18) | Moderate |
| Sota 400mg: Pla | 6.08 (2.10, 17.60) | Moderate^1^ | 0.01 (0.00, 1318640.45) | Moderate^3^ | 5.96 (2.06, 17.20) | Moderate |
| Sota 200mg: Sota 400mg | 0.65 (0.28, 1.49) | Moderate^1^ | 4.78 (0.02, 1125.22) | Moderate^3^ | 0.68 (0.30, 1.54) | Low^5^ |
| Cana 100mg: Dapa 10mg | - | - | 5.13 (0.25, 107.33) | Moderate^2^ | 5.13 (0.25, 107.33) | Moderate^7^ |
| Cana 100mg: Dapa 5mg | - | - | 4.41 (0.21, 92.47) | Moderate^2^ | 4.41 (0.21, 92.47) | Moderate^7^ |
| Cana 100mg: Empa 10mg | - | - | 4.22 (0.20, 88.00) | Moderate^2^ | 4.22 (0.20, 88.00) | Moderate^7^ |
| Cana 100mg: Empa 25mg | - | - | 5.75 (0.27, 121.41) | Moderate^2^ | 5.75 (0.27, 121.41) | Moderate^7^ |
| Cana 100mg: Empa 5mg | - | - | 3.78 (0.05, 299.57) | Moderate^2^ | 3.78 (0.05, 299.57) | Moderate^7^ |
| Cana 100mg: Ipra 100mg | - | - | 12.83 (0.11, 1471.83) | High^4^ | 12.83 (0.11, 1471.83) | High^7^ |
| Cana 100mg: Ipra 50mg | - | - | 17.29 (0.30, 982.69) | Moderate^2^ | 17.29 (0.30, 982.69) | Moderate^7^ |
| Cana 100mg: Sota 200mg | - | - | 2.80 (0.12, 66.46) | Moderate^2^ | 2.80 (0.12, 66.46) | Moderate^7^ |
| Cana 100mg: Sota 400mg | - | - | 1.90 (0.09, 41.96) | Moderate^2^ | 1.90 (0.09, 41.96) | Low^5^ |
| Cana 300mg: Dapa 10mg | - | - | 7.30 (0.36, 148.21) | Moderate^2^ | 7.30 (0.36, 148.21) | Moderate^7^ |
| Cana 300mg: Dapa 5mg | - | - | 6.28 (0.31, 127.69) | Moderate^2^ | 6.28 (0.31, 127.69) | Moderate^7^ |
| Cana 300mg: Empa 10mg | - | - | 6.01 (0.30, 121.50) | Moderate^2^ | 6.01 (0.30, 121.50) | Moderate^7^ |
| Cana 300mg: Empa 25mg | - | - | 8.19 (0.40, 167.65) | Moderate^2^ | 8.19 (0.40, 167.65) | Moderate^7^ |
| Cana 300mg: Empa 5mg | - | - | 5.38 (0.07, 417.57) | Moderate^2^ | 5.38 (0.07, 417.57) | Moderate^7^ |
| Cana 300mg: Ipra 100mg | - | - | 18.27 (0.16, 2054.97) | High^4^ | 18.27 (0.16, 2054.97) | High^7^ |
| Cana 300mg: Ipra 50mg | - | - | 24.62 (0.44, 1367.34) | Moderate^2^ | 24.62 (0.44, 1367.34) | Moderate^7^ |
| Cana 300mg: Sota 200mg | - | - | 3.99 (0.17, 91.88) | Moderate^2^ | 3.99 (0.17, 91.88) | Moderate^7^ |
| Cana 300mg: Sota 400mg | - | - | 2.71 (0.13, 57.97) | Moderate^2^ | 2.71 (0.13, 57.97) | Moderate^7^ |
| Dapa 10mg: Empa 10mg | - | - | 0.82 (0.23, 2.89) | Moderate^3^ | 0.82 (0.23, 2.89) | Moderate^7^ |
| Dapa 10mg: Empa 25mg | - | - | 1.12 (0.31, 4.05) | Moderate^3^ | 1.12 (0.31, 4.05) | Low^5^ |
| Dapa 10mg: Empa 5mg | - | - | 0.74 (0.02, 21.80) | Moderate^3^ | 0.74 (0.02, 21.80) | Low^5^ |
| Dapa 10mg: Ipra 100mg | - | - | 2.50 (0.05, 117.83) | Moderate^2^ | 2.50 (0.05, 117.83) | Low^5^ |
| Dapa 10mg: Ipra 50mg | - | - | 3.37 (0.18, 64.08) | Moderate^3^ | 3.37 (0.18, 64.08) | Moderate^7^ |
| Dapa 10mg: Sota 200mg | - | - | 0.55 (0.12, 2.55) | Moderate^3^ | 0.55 (0.12, 2.55) | Low^5^ |
| Dapa 10mg: Sota 400mg | - | - | 0.37 (0.09, 1.48) | Moderate^3^ | 0.37 (0.09, 1.48) | Low^5^ |
| Dapa 5mg: Empa 10mg | - | - | 0.96 (0.27, 3.38) | Moderate^3^ | 0.96 (0.27, 3.38) | Low^5^ |
| Dapa 5mg: Empa 25mg | - | - | 1.31 (0.36, 4.74) | Moderate^3^ | 1.31 (0.36, 4.74) | Low^5^ |
| Dapa 5mg: Empa 5mg | - | - | 0.86 (0.03, 25.41) | Moderate^3^ | 0.86 (0.03, 25.41) | Low^5^ |
| Dapa 5mg: Ipra 100mg | - | - | 2.91 (0.06, 137.31) | Moderate^2^ | 2.91 (0.06, 137.31) | Low^5^ |
| Dapa 5mg: Ipra 50mg | - | - | 3.92 (0.21, 74.72) | Moderate^3^ | 3.92 (0.21, 74.72) | Moderate^7^ |
| Dapa 5mg: Sota 200mg | - | - | 0.64 (0.14, 2.98) | Moderate^3^ | 0.64 (0.14, 2.98) | Low^5^ |
| Dapa 5mg: Sota 400mg | - | - | 0.43 (0.11, 1.73) | Moderate^3^ | 0.43 (0.11, 1.73) | Low^5^ |
| Empa 10mg: Empa 5mg | - | - | 0.89 (0.03, 26.43) | Moderate^3^ | 0.89 (0.03, 26.43) | Low^5^ |
| Empa 10mg: Ipra 100mg | - | - | 3.04 (0.06, 142.92) | Moderate^2^ | 3.04 (0.06, 142.92) | Low^5^ |
| Empa 10mg: Ipra 50mg | - | - | 4.10 (0.22, 77.67) | Moderate^3^ | 4.10 (0.22, 77.67) | Moderate^7^ |
| Empa 10mg: Sota 200mg | - | - | 0.66 (0.14, 3.08) | Moderate^3^ | 0.66 (0.14, 3.08) | Low^5^ |
| Empa 10mg: Sota 400mg | - | - | 0.45 (0.11, 1.79) | Moderate^3^ | 0.45 (0.11, 1.79) | Low^5^ |
| Empa 25mg: Empa 5mg | - | - | 0.66 (0.02, 19.57) | Moderate^3^ | 0.66 (0.02, 19.57) | Low^5^ |
| Empa 25mg: Ipra 100mg | - | - | 2.23 (0.05, 105.70) | Moderate^2^ | 2.23 (0.05, 105.70) | Low^5^ |
| Empa 25mg: Ipra 50mg | - | - | 3.00 (0.16, 57.60) | Moderate^3^ | 3.00 (0.16, 57.60) | Low^5^ |
| Empa 25mg: Sota 200mg | - | - | 0.49 (0.10, 2.31) | Moderate^3^ | 0.49 (0.10, 2.31) | Low^5^ |
| Empa 25mg: Sota 400mg | - | - | 0.33 (0.08, 1.35) | Moderate^3^ | 0.33 (0.08, 1.35) | Low^5^ |
| Empa 5mg: Ipra 100mg | - | - | 3.40 (0.02, 490.71) | Moderate^3^ | 3.40 (0.02, 490.71) | Low^5^ |
| Empa 5mg: Ipra 50mg | - | - | 4.58 (0.06, 340.21) | Moderate^3^ | 4.58 (0.06, 340.21) | Low^5^ |
| Empa 5mg: Sota 200mg | - | - | 0.74 (0.02, 24.61) | Moderate^3^ | 0.74 (0.02, 24.61) | Low^5^ |
| Empa 5mg: Sota 400mg | - | - | 0.50 (0.02, 15.65) | Moderate^3^ | 0.50 (0.02, 15.65) | Low^5^ |
| Ipra 100mg: Sota 200mg | - | - | 0.22 (0.00, 11.36) | Moderate^2^ | 0.22 (0.00, 11.36) | Low^5^ |
| Ipra 100mg: Sota 400mg | - | - | 0.15 (0.00, 7.28) | Moderate^2^ | 0.15 (0.00, 7.28) | Low^5^ |
| Ipra 50mg: Sota 200mg | - | - | 0.16 (0.01, 3.50) | Moderate^3^ | 0.16 (0.01, 3.50) | Low^5^ |
| Ipra 50mg: Sota 400mg | - | - | 0.11 (0.01, 2.21) | Moderate^3^ | 0.11 (0.01, 2.21) | Low^5^ |

**Footnote:**

1, Risk of bias; 2, Contributing direct evidence of high or moderate quality; 3, Contributing direct evidence of moderate quality; 4, Contributing direct evidence of high quality; 5. Imprecision.

SGLT-2i: Sodium-glucose co-transporter-2 inhibitors, Cana: Canagliflozin, Dapa: Dapagliflozin, Ipra: Ipragliflozin,Empa: Empagliflozin, Sota: Sotagliflozin,Pla: Placebo, DKA: diabetic ketoacidosis

## Supplementary Table 7. Results of sensitivity analyses for different doses of SGLT-2i

### **7.1 Exclusion of studies with fewer than 100 participants**

Outcome: The risk of DKA (odds ratio; 95% confidence interval)

| Cana 100mg |  |  |  |  |  |  |  |  |  |
| --- | --- | --- | --- | --- | --- | --- | --- | --- | --- |
| 0.70 (0.22,2.28) | Cana 300mg |  |  |  |  |  |  |  |  |
| 4.65 (0.22,100.30) | 6.62 (0.32,138.54) | Dapa 10mg |  |  |  |  |  |  |  |
| 3.95 (0.18,84.87) | 5.62 (0.27,117.22) | 0.85 (0.44,1.64) | Dapa 5mg |  |  |  |  |  |  |
| 3.74 (0.18,79.29) | 5.33 (0.26,109.50) | 0.81 (0.21,3.15) | 0.95 (0.24,3.68) | Empa 10mg |  |  |  |  |  |
| 5.19 (0.24,111.46) | 7.39 (0.36,153.94) | 1.12 (0.28,4.50) | 1.32 (0.33,5.26) | 1.39 (0.67,2.87) | Empa 25mg |  |  |  |  |
| 21.98 (0.17,2922.87) | 31.30 (0.24,4083.34) | 4.73 (0.08,273.10) | 5.57 (0.10,320.84) | 5.87 (0.10,334.24) | 4.23 (0.07,243.49) | Ipra 50mg |  |  |  |
| 11.33 (0.62,207.19) | 16.13 (0.91,285.68) | 2.44 (0.90,6.58) | **2.87 (1.07,7.67)** | **3.02 (1.19,7.70)** | 2.18 (0.82,5.79) | 0.52 (0.01,26.29) | Pla |  |  |
| 1.95 (0.08,48.07) | 2.77 (0.12,66.48) | 0.42 (0.08,2.25) | 0.49 (0.09,2.63) | 0.52 (0.10,2.70) | 0.37 (0.07, 1.99) | 0.09 (0.00,5.67) | 0.17 (0.04, 0.67) | Sota 200mg |  |
| 1.27 (0.05,29.47) | 1.81 (0.08,40.73) | 0.27 (0.06,1.30) | 0.32 (0.07,1.52) | 0.34 (0.07,1.55) | 0.24 (0.05,1.15) | 0.06 (0.00,3.52) | 0.11 (0.03, 0.37) | 0.65 (0.29,1.48) | Sota 400mg |

**Footnote:** Cana: Canagliflozin, Dapa: Dapagliflozin, Empa: Empagliflozin, Ipra: Ipragliflozin, Pla: Placebo,Sota:Sotagliflozin

### **7.2 Exclusion of studies with treatment duration <12 weeks**

Outcome: The risk of DKA (odds ratio; 95% confidence interval)

| Cana 100mg |  |  |  |  |  |  |  |  |  |
| --- | --- | --- | --- | --- | --- | --- | --- | --- | --- |
| 0.70 (0.22,2.28) | Cana 300mg |  |  |  |  |  |  |  |  |
| 4.55 (0.21,96.80) | 6.48 (0.31,133.69) | Dapa 10mg |  |  |  |  |  |  |  |
| 3.88 (0.18,82.80) | 5.53 (0.27,114.36) | 0.85 (0.44,1.64) | Dapa 5mg |  |  |  |  |  |  |
| 3.74 (0.18,79.29) | 5.33 (0.26,109.50) | 0.82 (0.22,3.11) | 0.96 (0.25,3.67) | Empa 10mg |  |  |  |  |  |
| 5.19 (0.24,111.46) | 7.39 (0.36,153.94) | 1.14 (0.29,4.45) | 1.34 (0.34,5.24) | 1.39 (0.67,2.87) | Empa 25mg |  |  |  |  |
| 21.98 (0.17,2922.87) | 31.30 (0.24, 4083.34) | 4.83 (0.08,275.78) | 5.66 (0.10,323.89) | 1.01 (0.03,30.19) | 4.23 (0.07,243.49) | Ipra 50mg |  |  |  |
| 11.33 (0.62,207.19) | 16.13 (0.91,285.68) | 2.49 (0.96,6.42) | **2.92 (1.12,7.58)** | **3.02 (1.19,7.70)** | 2.18 (0.82,5.79) | 0.52 (0.01,26.29) | Pla |  |  |
| 2.81 (0.12,67.83) | 4.00 (0.17,93.79) | 0.62 (0.12,3.09) | 0.72 (0.14,3.63) | 0.75 (0.16,3.58) | 0.54 (0.11,2.75) | 0.13 (0.00, 8.04) | 0.25 (0.07,0.91) | Sota 200mg |  |
| 1.90 (0.08,43.04) | 2.71 (0.12,59.48) | 0.42 (0.10,1.83) | 0.49 (0.11,2.15) | 0.51 (0.12,2.09) | 0.37 (0.08,1.63) | 0.09 (0.00, 5.18) | 0.17 (0.05, 0.52) | 0.68 (0.30,1.54) | Sota 400mg |

**Footnote:** Cana: Canagliflozin, Dapa: Dapagliflozin, Empa: Empagliflozin, Ipra: Ipragliflozin, Pla: Placebo,Sota:Sotagliflozin

### **7.3 Exclusion of studies without** **insulin-control**

Outcome: The risk of DKA (odds ratio; 95% confidence interval)

| Cana 100mg |  |  |  |  |  |  |  |  |  |  |  |
| --- | --- | --- | --- | --- | --- | --- | --- | --- | --- | --- | --- |
| 0.70 (0.22,2.28) | Cana 300mg |  |  |  |  |  |  |  |  |  |  |
| 5.03 (0.24,105.37) | 7.16 (0.35,145.50) | Dapa 10mg |  |  |  |  |  |  |  |  |  |
| 4.50 (0.21,94.55) | 6.41 (0.31,130.56) | 0.90 (0.46,1.74) | Dapa 5mg |  |  |  |  |  |  |  |  |
| 4.22 (0.20,88.00) | 6.01 (0.30,121.50) | 0.84 (0.24,2.95) | 0.94 (0.26,3.32) | Empa 10mg |  |  |  |  |  |  |  |
| 5.75 (0.27,121.41) | 8.19 (0.40,167.65) | 1.14 (0.32,4.14) | 1.28 (0.35,4.65) | 1.36 (0.68,2.76) | Empa 25mg |  |  |  |  |  |  |
| 3.78 (0.05,299.57) | 5.38 (0.07,417.57) | 0.75 (0.03,22.26) | 0.84 (0.03,24.91) | 0.89 (0.03,26.43) | 0.66 (0.02,19.57) | Empa 5mg |  |  |  |  |  |
| 12.83 (0.11,1471.83) | 18.27 (0.16, 2054.97) | 2.55 (0.05,120.28) | 2.85 (0.06,134.61) | 3.04 (0.06,142.92) | 2.23 (0.05,105.70) | 3.40 (0.02,490.71) | Ipra 100mg |  |  |  |  |
| 17.29 (0.30, 982.69) | 24.62 (0.44,1367.34) | 3.44 (0.18,65.43) | 3.84 (0.20,73.27) | 4.10 (0.22,77.67) | 3.00 (0.16,57.60) | 4.58 (0.06, 340.21) | 1.35 (0.03,56.92) | Ipra 50mg |  |  |  |
| 11.33 (0.62,207.19) | 16.13 (0.91,285.68) | 2.25 (0.92,5.53) | **2.52 (1.02,6.22)** | **2.68 (1.11,6.49)** | 1.97 (0.78,4.94) | 3.00 (0.11,78.81) | 0.88 (0.02,37.42) | 0.65 (0.04,10.83) | Pla |  |  |
| 2.80 (0.12,66.46) | 3.99 (0.17,91.88) | 0.56 (0.12,2.61) | 0.62 (0.13,2.93) | 0.66 (0.14,3.08) | 0.49 (0.10,2.31) | 0.74 (0.02 , 24.61) | 0.22 (0.00,11.36) | 0.16 (0.01,3.50) | 0.25 (0.07.0.87) | Sota 200mg |  |
| 1.90 (0.09,41.96) | 2.71 (0.13,57.97) | 0.38 (0.09, 1.52) | 0.42 (0.10,1.70) | 0.45 (0.11,1.79) | 0.33 (0.08,1.35) | 0.50 (0.02, 15.65) | 0.15 (0.00,7.28) | 0.11 (0.0,2.21) | 0.17 (0.06. 0.48) | 0.68 (0.30,1.54) | Sota 400mg |

**Footnote:** Cana: Canagliflozin, Dapa: Dapagliflozin, Empa: Empagliflozin, Ipra: Ipragliflozin, Pla: Placebo,Sota:Sotagliflozin

### **7.4 This analysis was estimated in a Bayesian framework**

Outcome: The risk of DKA (odds ratio; 95% confidence interval)

| Cana100mg |  |  |  |  |  |  |  |  |  |  |  |
| --- | --- | --- | --- | --- | --- | --- | --- | --- | --- | --- | --- |
| 0.69 (0.07, 6.17) | Cana300mg |  |  |  |  |  |  |  |  |  |  |
| **51247756.66 (4.86, 1.77e+26)** | **76909725.69 (7.13, 2.30e+26)** | Dapa10mg |  |  |  |  |  |  |  |  |  |
| **43825881.56 (3.94, 1.36e+26)** | **68156479.56 (6.01, 1.83e+26)** | 0.85 (0.23, 3.12) | Dapa5mg |  |  |  |  |  |  |  |  |
| **78736636.41 (6.81, 1.99e+26)** | 1**22802952.16 (8.41, 2.63e+26)** | **1.39 (0.2, 23.85)** | **1.65 (0.24, 29.08)** | Empa10mg |  |  |  |  |  |  |  |
| 1**04673854.62 (9.55, 2.77e+26)** | **157442982.23 (11.64, 3.89e+26)** | **1.94 (0.27, 33.63)** | **2.27 (0.32, 43.51)** | **1.39 (0.31, 6.26)** | Empa25mg |  |  |  |  |  |  |
| 0 (0, 12358753674300272640) | 0 (0, 16611550034624407552) | 0 (0, 1.53) | 0 (0, 1.61) | 0 (0, 1.03) | 0 (0, 0.6) | Empa5mg |  |  |  |  |  |
| **3577082.79 (0,7.43e+36)** | **5003632.78 (0,1.29e+37)** | 0(0,444451672  676424) | 0(0,603024059  242860) | 0(0,436247285647964) | 0(0,301539955324214) | **87394385755.61 (0, 8.64e+39)** | Ipra100mg |  |  |  |  |
| **4453136077645861376 (0, 2.37e+54)** | **6638941042732149760 (0, 3.88e+54)** | **74799855.63 (0, 7.38e+41)** | **84691829.11 (0, 9.628e+41)** | **54622335.6 (0, 4.0e+41)** | **40762188.08 (0, 3.16e+41)** | **2.30321493927832e+21 (0, 3.77e+77)** | **1346310.4 (0, 1.11e+59)** | Ipra50mg |  |  |  |
| **238580208.7 (25.71, 6.87e+26)** | **359691821.09 (33.53, 9.27e+26)** | **4.46 (1.23, 42.42)** | **5.28 (1.41, 52.49)** | **3.18 (0.67, 16.86)** | **2.26 (0.46, 12.47)** | **9706552512540904 (4.01, 1.48e+39)** | **5260.87 (0, 3.63e+35)** | 0 (0, 5.13e+22) | Pla |  |  |
| **49297461.64 (4.09, 1.75e+26)** | **74334630.57 (5.52, 2.27e+26)** | 0.84 (0.07, 27.26) | 0.98 (0.09, 34.76) | 0.57 (0.05, 11.57) | 0.41 (0.04, 8.44) | **2279679369097896 (0.74, 1.97e+38)** | **1002.53 (0, 6.27e+34)** | 0 (0, 1.24e+22) | 0.18 (0.02, 1.87) | Sota200mg |  |
| **18260822.41 (2.06, 6.89006833315894e+25)** | **28442153.03 (2.44, 8.95e+25)** | 0.36 (0.03, 4.46) | 0.42 (0.04, 5.56) | 0.25 (0.02, 2.13) | 0.18 (0.01, 1.52) | **869774511339210 (0.28, 9.01e+37)** | **380.99 (0, 2.89e+34)** | 0 (0, 5.00e+21) | 0.08 (0.01, 0.32) | 0.44 (0.04, 1.45) | Sota400mg |

## Footnote: Cana: Canagliflozin, Dapa: Dapagliflozin, Empa: Empagliflozin, Ipra: Ipragliflozin, Pla: Placebo,Sota: Sotagliflozin.

## Supplementary Figure 1. Inconsistency (incoherence) assessments for different doses of SGLT-2i


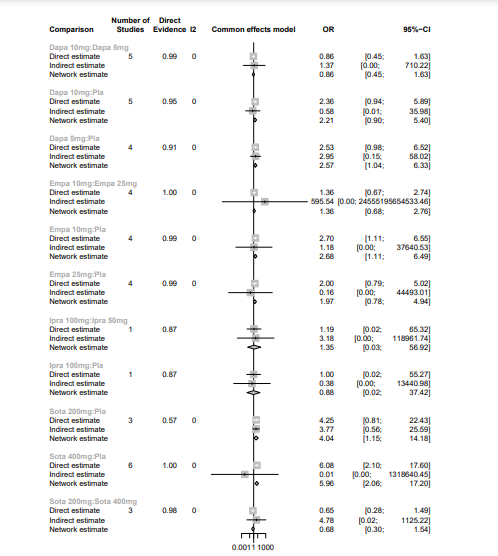


## Supplementary Figure 2. Intransitivity assessments-age at baseline


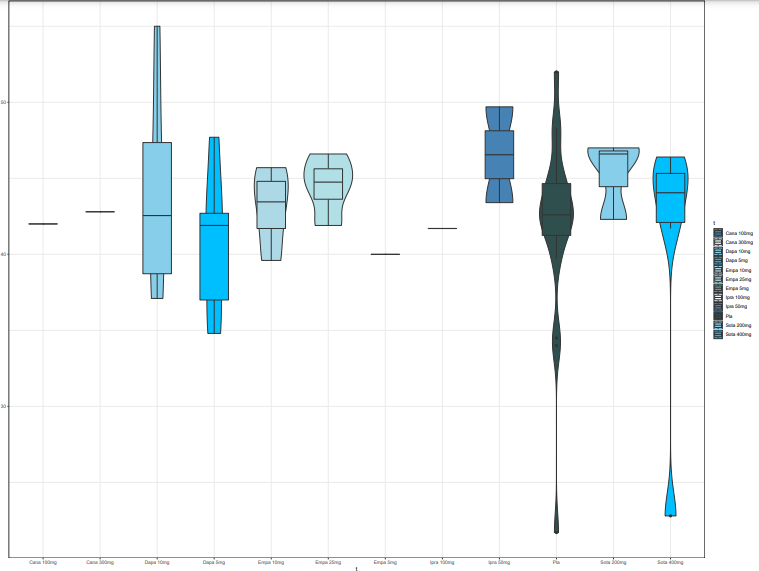


## Supplementary Figure 3. Intransitivity assessments-BMI at baseline


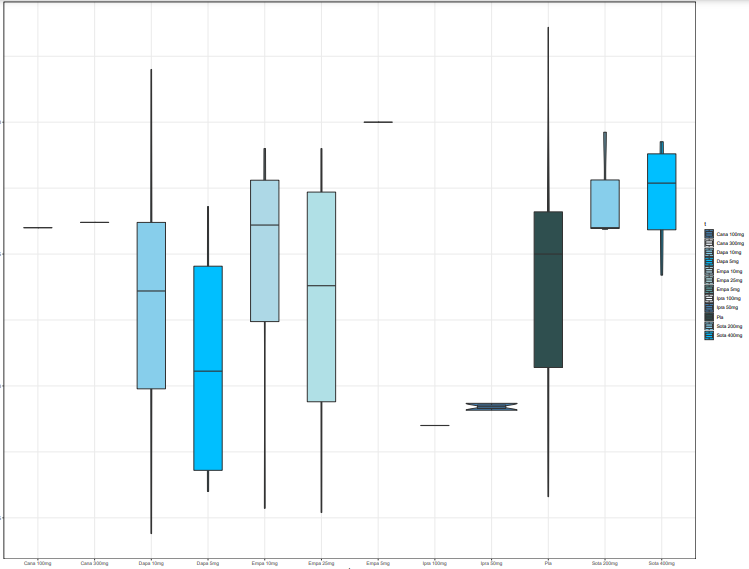


## Supplementary Figure 4. Intransitivity assessments-HbA1c at baseline


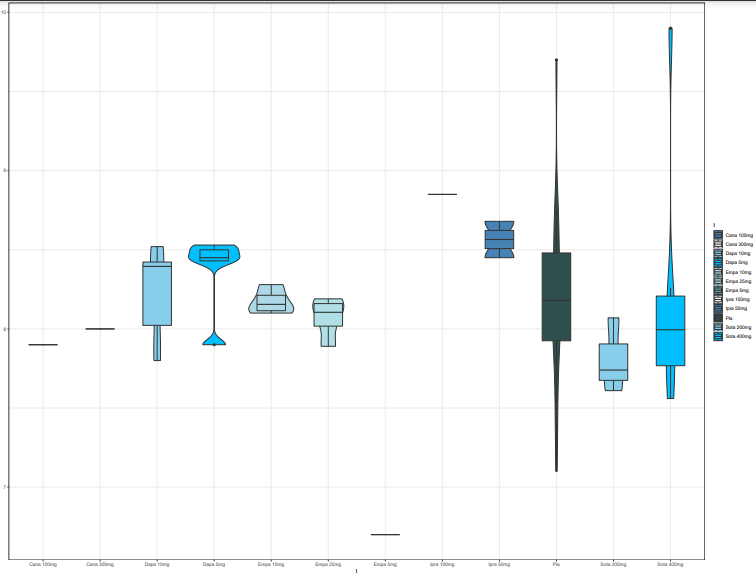


## Supplementary Figure 5. Funnel plot for different kinds of active antidiabetic drugs


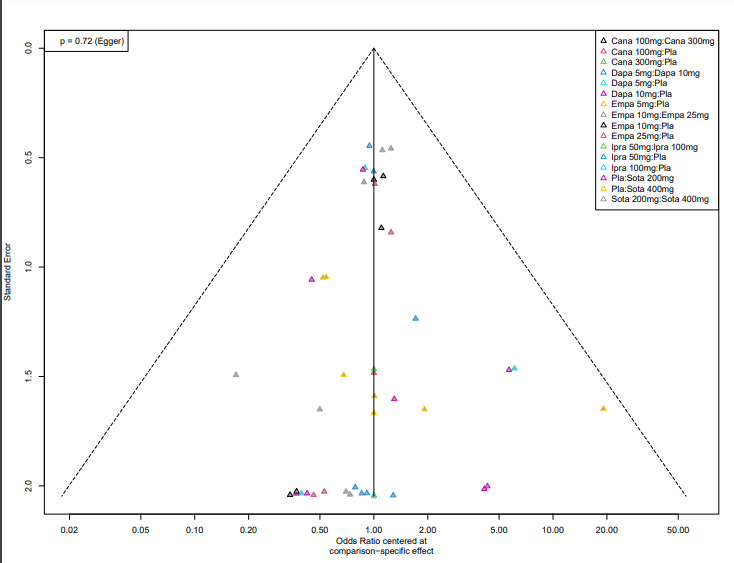

Supplement: Supplementary file 1 [file DataSheet1.docx]
